# Long Term Diversity and Distribution of Non-photosynthetic Cyanobacteria in Peri-Alpine Lakes

**DOI:** 10.3389/fmicb.2018.03344

**Published:** 2019-01-14

**Authors:** Marie-Eve Monchamp, Piet Spaak, Francesco Pomati

**Affiliations:** ^1^Department of Aquatic Ecology, Swiss Federal Institute of Aquatic Science and Technology, Eawag, Dübendorf, Switzerland; ^2^Swiss Federal Institute of Technology, Institute of Integrative Biology, Zurich, Switzerland

**Keywords:** Melainabacteria, ML635J-21, Sericytochromatia, metabarcoding, sedimentary DNA, distance-decay relationship, meta-community, Anthropocene

## Abstract

The phylum Cyanobacteria comprises a non-photosynthetic lineage. The diversity and distribution of non-photosynthetic cyanobacteria (NCY) across aquatic environments are currently unknown, including their ecology. Here, we report about composition and phylogenetic diversity of two clades of NCY in ten lakes of the European peri-Alpine region, over the past ∼100 years. Using 16S rDNA sequences obtained from dated sediment cores, we found almost equal proportion of taxa assigned to Melainabacteria and the deepest-branching group Sericytochromatia (ML635J-21) (63 total detected taxa). The topology of our reconstructed phylogenies reflected evolutionary relationships expected from previous work, that is, a clear separation between the deepest branching Sericytochromatia, the Melainabacteria, and the photosynthetic cyanobacteria clades. While different lakes harbored distinct NCY communities, the diversity of NCY assemblages within and between lakes (alpha and beta diversity) did not significantly change over the last century. This is in contrast with what was previously reported for photosynthetic cyanobacteria. Unchanged community phylogenetic similarity over geographic distance indicated no dispersal limitation of NCY at the regional scale. Our results solicit studies linking in-lake environmental factors to the composition of these microorganisms’ communities, whose assembly appeared not to have been influenced by large-scale anthropogenic environmental changes. This is the first attempt to study the diversity and distribution of NCY taxa across temperate lakes. It provides a first step towards understanding their distribution and ecological function in pelagic aquatic habitats, where these organisms seem to be prevalent.

## Introduction

Cyanobacteria are a highly diverse group of gram-negative prokaryotes that have colonized a wide range of environments, from desert crusts to fresh and marine waters, and from the tropics to the poles (Whitton and Potts, 2002). They have played a crucial role in modifying the Earth’s atmosphere through the process of oxygenic photosynthesis, which enabled the evolution of life in more complex forms (Fischer et al., 2016b). Cyanobacteria have been studied for decades, and their diversity has been described both morphologically (Rippka et al., 1979; Komárek et al., 2014) and genetically (Shih et al., 2013). Cyanobacteria are often considered a nuisance in aquatic ecosystems as they can form large blooms and decrease water quality, ecosystem functions and ecosystem services (Rigosi et al., 2014).

With the development of molecular techniques allowing the investigation of non-cultivable organisms, an unexpected diversity of cyanobacteria was unveiled in many ecosystems (Sogin et al., 2006; Di Rienzi et al., 2013; Soo et al., 2014; Meola et al., 2015). Genome sequencing recently revealed important information about a clade of non-photosynthetic prokaryotes closely related to the Cyanobacteria. The name Melainabacteria was proposed (Di Rienzi et al., 2013), because several representatives of this group have been found in aphotic environments. They were first thought to constitute a sister-phylum of the Cyanobacteria (Di Rienzi et al., 2013), but additional genomic information confirmed the position of Melainabacteria as a sister-clade of photosynthetic cyanobacteria and as part of the same phylum (Soo et al., 2014). Based on genomic evidence, a re-classification has been proposed for the phylum Cyanobacteria, with the class-level lineages Oxyphotobacteria (all cyanobacteria capable of photosynthesis) and Melainabacteria, as well as a third class called ML635J-21 (Soo et al., 2014), for which the name Sericytochromatia was recently proposed (Soo et al., 2017). Sericytochromatia is the most basal lineage and forms a paraphyletic group that is the ancestor of both Melainabacteria and photosynthetic cyanobacteria (Fischer et al., 2016a; Soo et al., 2017). After diverging from Melainabacteria, the Oxyphotobacteria developed oxygenic photosynthesis around 2.4–2.35 billion years ago based on molecular clock estimates (see Shih et al., 2016; Soo et al., 2017) and geological data (Fischer et al., 2016a).

For ease of discussion, we hereafter refer to class Oxyphotobacteria as photosynthetic cyanobacteria, and we use the nomenclature proposed by (Soo et al., 2014, 2017) for the class Melainabacteria, wherein the following orders are included: Gastranaerophilales (YS2), Obscuribacterales (mle1-12), Caenarcaniphilales (ACD20), and Vampirovibrionales (SM1D11). Because there is only a hand-full of Melainabacteria and Sericytochromatia sequenced genomes (Soo et al., 2017), the metabolism, functions, and ecological role of these organisms are not fully known. Representatives of Melainabacteria and Sericytochromatia have been found both in photic and aphotic environments, such as subsurface ground water (Di Rienzi et al., 2013), lake water and algal biofilms (Soo et al., 2017; Salmaso et al., 2018), marine and lacustrine sediment (Ley et al., 2005), animal and human feces (Soo et al., 2014) and guts (Ley et al., 2005). A list of non-photosynthetic cyanobacteria (NCY) representatives with their associated environments is presented in Table 1.

**Table 1:** List of Melainabacteria and Sericytochromatia representatives and the type of environment in which they have been found.

Class	Order	Environment	Reference
Melainabacteria	Gastranaerophilales	Human fecal samples	Qin et al., 2010; Soo et al., 2014
	Gastranaerophilales	Koala fecal samples	Soo et al., 2014
	Gastranaerophilales	Soil sample	McGorum et al., 2015
	Obscuribacterales	Activated sludge from a batch (aerobic) reactor performing enhanced biological phosphorus removal (EBPR)	Soo et al., 2014
	Obscuribacterales	Plant washing; horse ileal content; soil sample	McGorum et al., 2015
	Obscuribacterales	Aquifer	Wrighton et al., 2012; Di Rienzi et al., 2013
	Caenarcaniphilales	Anaerobic reactor treating a high-strength organic wastewater	Soo et al., 2014
	Caenarcaniphilales	Freshwater lakes (epilimnion)	Soo et al., 2017; Salmaso et al., 2018
	Vampirovibrionales Vampirovibrionales	*Chlorella vulgaris* culture *Chlorella* in commercial ponds	Soo et al., 2015; Ganuza et al., 2016
	Vampirovibrionales	Multiisolate	Soo et al., 2017
Sericytochromatia (ML635J-21)	NA	Plant washing; soil sample; marine and lacustrine sediments	McGorum et al., 2015
	NA	Freshwater lakes (epilimnion) marine surface water samples (uncultured marine bacteria)	Gies et al., 2014; Salmaso et al., 2018
	NA	Subsurface groundwater	Soo et al., 2017

All sequenced genomes of Melainabacteria confirm that they completely lack the photosynthesis apparatus, supporting the hypothesis that the acquisition of the photosystem in photosynthetic cyanobacteria happened after divergence from the non-photosynthetic Melainabacteria (Soo et al., 2014, 2017). All representatives of Melainabacteria are thought to be able to perform fermentation and some are thought to be capable of anaerobic and aerobic respiration (Di Rienzi et al., 2013; Soo et al., 2014, 2017). Some Melainabacteria in the Caenarcaniphilales, Vampirovibrionales, and Obscuribacterales orders are also capable of aerobic respiration under both high and low oxygen conditions (Soo et al., 2017). This ability has been acquired after the evolution of oxygenic photosynthesis in the sister-clade photosynthetic cyanobacteria (Shih et al., 2016; Soo et al., 2017). The Gastranaerophilales are found in human and other animal guts and although their exact role is unknown, they are thought to have a beneficial effect for their hosts by aiding digestion and as a source of vitamins B and K (Di Rienzi et al., 2013). Some Gastranaerophilales are flagellated, but other representatives appear to have only a subset of the necessary genes for encoding a functional flagellum (Soo et al., 2014). The Gastranaerophilales and Caenarcaniphilales seem to lack the genes for anaerobic and aerobic respiration, suggesting that they support their metabolism by fermentation only (Soo et al., 2014). *Vampirovibrio chlorellavorus* (previously assigned to genus *Bdellovibrio*) is a predatory bacterium that was originally described in 1972, and which was first suggested being a member of the Deltaproteobacteria (Gromov and Mamkaeva, 1972). This bacterium was recently isolated from a 36-year old co-culture sample of *Chlorella vulgaris* lyophilised cells (NCBI 11383) (Coder and Starr, 1978) and whole genome sequencing confirmed the position of *Vampirovibrio* in the class Melainabacteria (order Vampirovibrionales) part of phylum Cyanobacteria (Soo et al., 2015). *V. chlorellavorus* predates on *Chlorella* cells and, consistently with other classes of Melainabacteria, it lacks the genes for carbon fixation and photosynthesis (Soo et al., 2014).

In this study, we used a dataset consisting of 16S rDNA sequences obtained from high-throughput amplicon sequencing of samples collected in sediment cores from ten lakes, spanning ∼100 years (from ∼1900 to present), to study the diversity and distribution of NCY over the Anthropocene in the European peri-Alpine region. Lake sediments are well known to constitute rich archives of information about freshwater pelagic communities, in terms of both species resting stages and biomolecules (Gregory-Eaves and Beisner, 2011). Sedimentary DNA (sedDNA) in particular has been instrumental in reconstructing past community composition of various plankton forms, such as bacteria, phyto- and zooplankton, as well as microbial eukaryotes (Domaizon et al., 2017). Cyanobacteria were found to be well preserved in deep hard-water lakes (Domaizon et al., 2013; Monchamp et al., 2016). We have demonstrated previously that the reconstruction of cyanobacterial community structure and phylogenetic diversity using sedDNA, amplification, and sequencing relates almost 1:1 to pelagic microscopic counts from lakes over the past 40 years (Monchamp et al., 2016). Here, we used 16S rDNA sequences from sedimentary archives to investigate changes in NCY prevalence and community composition over time across our panel of ten lakes. Since we were only interested in the diversity of environmentally associated NCY, we present the composition of all groups of NCY, but exclude the gut-associated Gastranaerophilales order from the diversity analyses. Additionally, we tested for changes in community similarity across lakes and geographic distances to infer patterns of NCY dispersal at the regional scale. Results are discussed in comparison with previously observed community changes in photosynthetic cyanobacterial assemblages in the same lake samples (Monchamp et al., 2018a).

## Materials and Methods

### Data Collection

We used the high-throughput MiSeq (Illumina) data from (Monchamp et al., 2018a), consisting of 16S rDNA cyanobacterial sequences obtained from sequencing of 107 DNA samples spanning over ∼100 years of sedimentary archives from 10 lakes located around the European Alps (Supplementary Tables S1, S2). These lakes have been chosen based on their geographic location, their eutrophication history, and because they cover a wide gradient of trophic levels and morphological characteristics (Supplementary Table S1). History of these lakes is well known due to monitoring programs carried out by various governmental and institutional agencies over the last two to six decades (Monchamp et al., 2018a). The lakes are located at altitudes between 194 and 463 m above sea level (mean = 369, median = 400.5), with differences between altitudes of less than 300m. Therefore, relative to their environmental change history and physico-chemical properties, we did not consider altitude as an important factor for our study. Due to the natural formation of annual varves over the last century in the deep hard-water lakes studied, dating could be performed at high (yearly) temporal resolution in most cases. In addition, the cores were dated based on lead and cesium radioisotope (^210^Pb, ^137^Cs) measurements (see Monchamp et al., 2018a).

The study is based on environmental DNA, i.e., both extracellular and intracellular DNA that is preserved in lake sediments. The raw sequences are publically available in the European Nucleotide Archive repository; https://www.ebi.ac.uk/ena under the project number PRJEB21329. The amplified region of approximately 400 bp in length (range = 370–415 bp, mean = 386.2 bp) covers part of the V3–V4 regions of the 16S gene. A large proportion of the sequences (almost 20%) were classified as part of the Cyanobacteria phylum, but did not belong to photosynthetic cyanobacteria. These sequences were highly related to the Melainabacteria and ML635J-21 (Sericytochromatia) groups in the Greengenes database (DeSantis et al., 2006). Here, we used this subset of sequence data to investigate the alpha and beta diversity of NCY in the ten lakes.

All details related to sediment sampling, sample processing, DNA extraction, PCR amplification, and sequencing are described in our previous work (Monchamp et al., 2016, 2018a). One or two cores from each lake were opened longitudinally and sediment samples were collected at various depths based on the age models. Sediments were then transferred to a clean lab facility where DNA was extracted using the PowerSoil DNA Isolation Kit (Mo Bio Laboratories). We performed two DNA extractions per layer and pooled the two extracts to increase homogeneity of sampling. This pooled sedDNA was used in PCR reactions (three separate PCR per sample) using the pair of cyanobacteria-specific primers CYA359-F and CYA784-R (Nübel et al., 1997; DeSantis et al., 2006) attached to individual tags of 10–12 nucleotides (Monchamp et al., 2018a). The purified extracts were then pooled in equimolar concentration in a single library which was sent to the sequencing facility (Fasteris, Geneva, Switzerland) for adaptor ligation and sequencing on an Illumina MiSeq platform.

### Sequence Data Processing

Briefly, the raw 16S rDNA sequences were quality controlled using the workflow developed at the Genetic Diversity Centre, ETH Zürich as described elsewhere (Monchamp et al., 2016, 2018a). After quality filtering, primer trimming and size selection, the amplicons were clustered into operational taxonomic units (OTUs) following the UPARSE workflow (Edgar, 2013), based on an abundance threshold of 5, and a minimum sequence similarity of 97%. The taxonomic assignment of OTUs was done with a confidence threshold of 89% based on the Greengenes database (DeSantis et al., 2006; Monchamp et al., 2016) using PyNAST (McDonald et al., 2012). The latter database was chosen because it was found to comprise a broad range of representatives in phylum Cyanobacteria, including the NCY groups (Monchamp et al., 2016). The alignment was finally imported in FastTree (Price et al., 2010) to infer a phylogeny based on maximum-likelihood.

**FIGURE 1:**
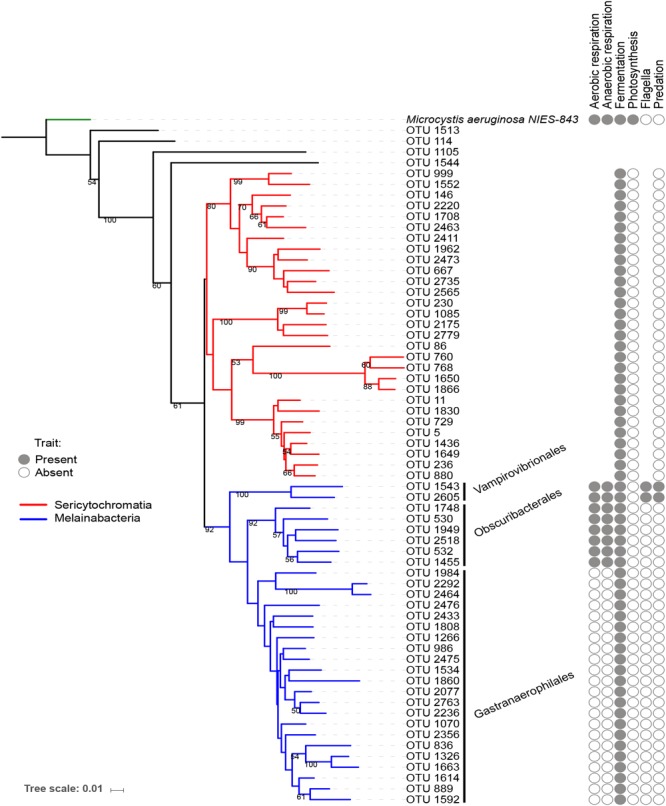
Neighbor joining phylogenetic tree based on all OTU reference sequences assigned to NCY in this study, with *Microcystis aeruginosa* (photosynthetic cyanobacteria) as outgroup. Bootstrap values >0.50 are shown. The clade in red was assigned to class Sericytochromatia, and taxa highlighted in blue are representatives of class Melainabacteria, which splits into three orders: Gastranaerophilales, Obscuribacterales, Vampirovibrionales, and Caenarcaniphilales (not represented). The table next to the phylogeny indicates whether representatives of the group have been found to possess (full circle), or lack (empty circle) a given functional trait (based on the available literature). Absence of circle signifies missing information.

**FIGURE 2:**
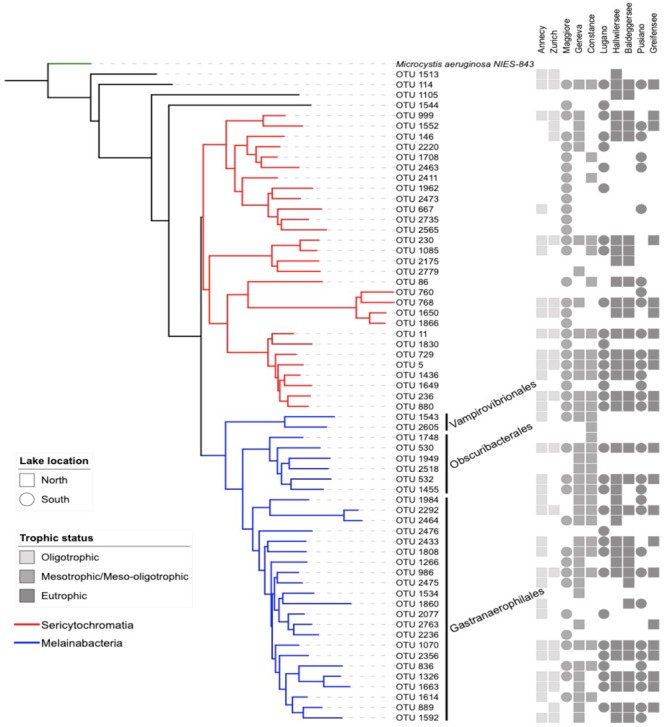
Neighbor joining phylogenetic tree based on all OTU reference sequences assigned to NCY in this study (same as Figure 1), with *M. aeruginosa* (photosynthetic cyanobacteria) as outgroup. The table next to the phylogeny summarizes the OTU composition in each lake (detected; full shape, not detected; no shape). The shades of gray refer to the lake’s trophic status and the shapes refer to the location of the lakes relative to the Alpine mountain range (square; north, circles; south).

The reference OTUs FASTA sequences, the tree file, and the taxonomic assignment file were imported in the software R (R Development Core Team, 2016) version 3.3.2, using the package “phyloseq” in Bioconductor (McMurdie and Holmes, 2013). The dataset was filtered to retain only the NCY OTUs. Of these, twenty-nine were assigned to the deepest-branching clade Sericytochromatia, thirty OTUs were assigned to Melainabacteria (two Vampirovibrionales, six Obscuribacterales, and twenty-two Gastranaerophilales), and four NCY OTUs were not assigned to a class. The trees in Figures 1, 2 are representations of all of the NCY OTUs recovered (63 in total). The phylogeny was inferred based on neighbor joining with bootstrap analysis (100 replicates) using the alignment program MAFFT (Katoh and Standley, 2013) and visualized and annotated with the online program iTOL (Letunic and Bork, 2016). OTUs that were found in less than two samples over the whole dataset were excluded from the diversity analyses to reduce biases associated to rare taxa and to possible sequencing errors.

A small number of physiological traits have been previously attributed to Melainabacteria and Sericytochromatia based on genome sequencing and analysis (Di Rienzi et al., 2013; Soo et al., 2014, 2017). We added information about respiration, photosynthesis, motility, and known predatory behavior to the phylogenetic tree of OTUs for reference (Figure 1). Note that this list of traits is an extrapolation of the information based on genome sequencing of a small subset of Melainabacteria representatives, thus the definitive presence or absence of the listed traits in the OTUs found in the present study cannot be confirmed.

### Compositional and Phylogenetic Diversity Analyses

For the diversity analyses, we used all OTUs assigned to Sericytochromatia, as well as the Melainabacteria representatives that are generally associated with environmental samples [i.e., the orders Vampirovibrionales (SM1D11) and Obscuribacterales (mle1-12)]. We excluded the order Gastranaerophilales that is associated with gut samples as it was considered irrelevant to the present study aiming at describing the diversity of NCY lake communities. Our dataset did not include representatives of the Caenarcaniphilales order.

Using this reduced dataset, we assessed the diversity and community composition of NCY within and across the 10 peri-Alpine lakes over ∼100 years. In order to estimate indices of alpha and beta diversity that can be used for comparisons, samples were rarefied to even sequencing depth (201 reads per sample) using the *rarefy_even_depth* function in “phyloseq.” This coverage retained a high number of samples, and was sufficient to cover a high percentage of OTU richness in the majority of samples (Supplementary Table S3 and Supplementary Figure S1). We used linear ordinary least square (OLS) regression to determine the significance of variation in the log-transformed rarefied OTU richness over time within the lakes. Hierarchical clustering of taxa for pattern detection was performed by calculating Euclidian distances on OTU prevalence in the lakes over time. The dendrogram was constructed by average linkage method and the color-coded image map produced in CIMminer (Weinstein et al., 1997).

For calculating beta diversity between communities across all lakes and between time periods, the rarefied samples were binned into 10-year blocks (to accommodate for the number of samples available at given years and the dating precision). A distance matrix based on phylogenetic dissimilarity (Unifrac distances) across all samples at each period was calculated. The “adonis” function in the “vegan” package (Oksanen et al., 2013) was used to apply PERMANOVA (Anderson, 2001) to verify temporal and spatial (lake and region) effects on the dissimilarity between groups. If a lake sediment core was sampled at two depths that were grouped in the same period of time (e.g., years 1992 and 1997), the pairwise distance between the two samples was excluded from the dissimilarity estimation (to remove the internal turnover effect within lakes). We used the package “Imap” version 1.32 for producing a matrix of pairwise geographic distances between lakes (Supplementary Table S2). This distance matrix was used for assessing the distance-decay relationship for all community phylogenies in each decade over the twentieth century. For comparison with the photosynthetic cyanobacteria communities, we used data from (Monchamp et al., 2018a,b). The data was insufficient to estimate the distance-decay relationship between NCY communities in the 1900s and the 2010s. Because the assumption of independence is violated when using multiple pairwise comparisons, the significance of the distance-decay curves at each time period was assessed by a Mantel (Mantel, 1967) permutational test (with 999 repetitions) between distance matrices using the package “ade4” for R.

## Results

### Composition and Phylogenetic Diversity of Non-photosynthetic Cyanobacteria

Our rarefied dataset of Sericytochromatia and Melainabacteria comprised of 63 taxa distributed in 66 samples. The neighbor joining tree of all 16S rDNA reference sequences (OTUs) recovered from the sediments of the ten peri-Alpine lakes is shown in Figure 1. The two lineages Sericytochromatia and Melainabacteria are well supported by the bootstrap values. Figure 2 shows the same phylogeny as in Figure 1 together with a table showing the distribution of the OTUs in each lake.

### Richness Change Over Time

**FIGURE 3:**
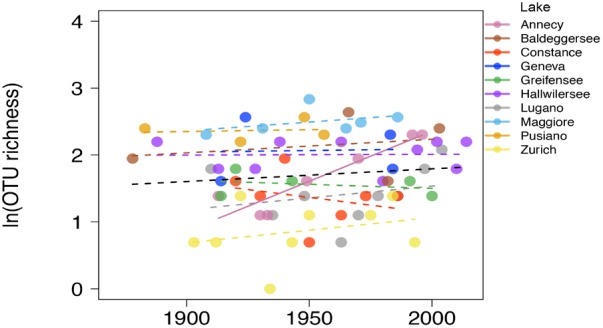
Natural log-transformed OTU richness of NCY associated with environmental samples over time in the ten lakes. The dashed lines show the non-significant linear fit for each lake, and the full line (Lake Annecy only) shows a significant OLS fit (*R*^2^ = 0.862, *p* = 0.003, *n* = 7). The OLS fit over all observations (*n* = 66) is not significant at the *p* = 0.05 level (black dashed line).

All the following analyses are based on the reduced dataset where the gut-associated lineage Gastranaerophilales, not considered relevant for the present environmental survey, was excluded. They comprised about 1/3 of all NCY taxa so, after exclusion of Gastranaerophilales, the final rarefied data comprised 40 taxa in 66 samples. The majority of OTUs (30) were assigned to class Sericytochromatia, and the Melainabacteria OTUs mostly belonged to the Obscuribacterales order (5 OTUs). A single OTU was assigned to Vampirovibrionales, and four OTUs were not assigned to a class. The richness of environmentally associated NCY OTUs did not increase significantly over the last century across the studied lakes (*n* = 66, *p* > 0.3; Figure 3). Lake Annecy was the only exception where the richness of NCY increased significantly over time (*R*^2^= 0.86, *p* = 0.003, *n* = 7).

### Temporal Changes in OTU Prevalence

**FIGURE 4:**
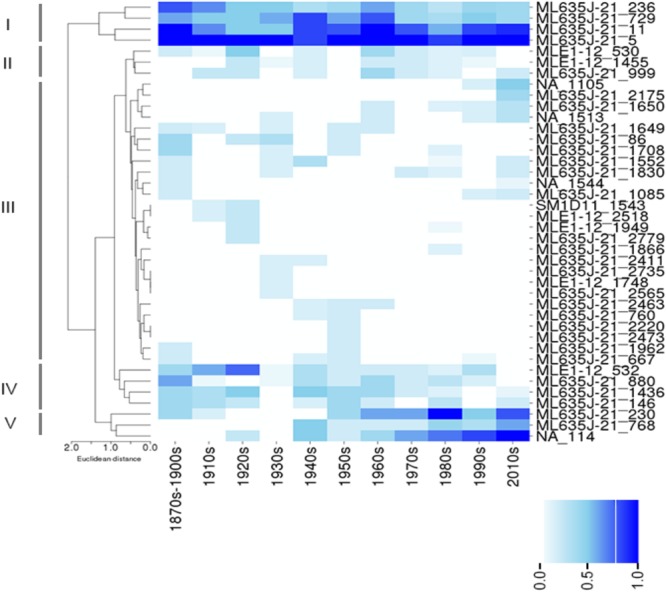
Color-coded map showing the prevalence (i.e., the proportion of lakes where an OTU was found at a given time period) of Sericytochromatia (ML635J-21), Obscuribacterales, and Vampirovibrionales in the lakes between the 1870s and the 2010s. Hierarchical clustering was based on Euclidian distances based on prevalence of OTUs and revealed five main clusters: I–V (see section “Results”). The absence of an OTU at a given time period is depicted by a white box, and the gradient of color is proportional to the prevalence of an OTU in the group of lakes sampled at a given time period.

Clustering of NCY OTUs highlights five groups (Figure 4). Cluster I comprises of four OTUs that were prevalent in the lakes at all time-periods. Representatives of this cluster are all affiliated to class Sericytochromatia. Cluster II shows a large number of OTUs that appear to be randomly distributed and that were never found in high prevalence across the ten lakes. Cluster III comprises of rare and isolated OTUs, and cluster IV highlights a group of OTUs that were generally more prevalent in older times compared to the last 3–4 decades. Most of the latter OTUs were also found in recent sediment, but they were less common across the region. They are mainly representatives of the Sericytochromatia class, and one OTU is assigned to Obscuribacterales. Finally, cluster V is composed of two Sericytochromatia representatives, and one unidentified OTU, which is most likely associated with class Sericytochromatia (ML635J-21) considering its position in the phylogeny (see OTU 114 in Figure 1). Interestingly, OTUs in Cluster V show the opposite pattern observed in Cluster IV: they became more common across the region since the 1940s (Figure 4).

### Temporal and Regional Change in Similarity

**FIGURE 5:**
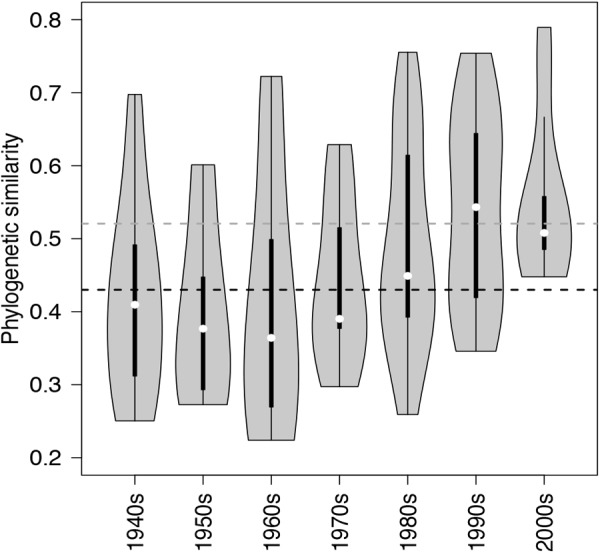
Violin plots showing the probability density of phylogenetic similarities (overall range, interquartile ranges and median – based on Unifrac) among lake communities of NCY at each decade from the 1940s to the 2000s. The horizontal dashed lines, representing the mean similarity value across all observations for each group of cyanobacteria (black for photosynthetic cyanobacteria from Monchamp et al. (2018a); dark gray for NCY), are shown for reference.

**FIGURE 6:**
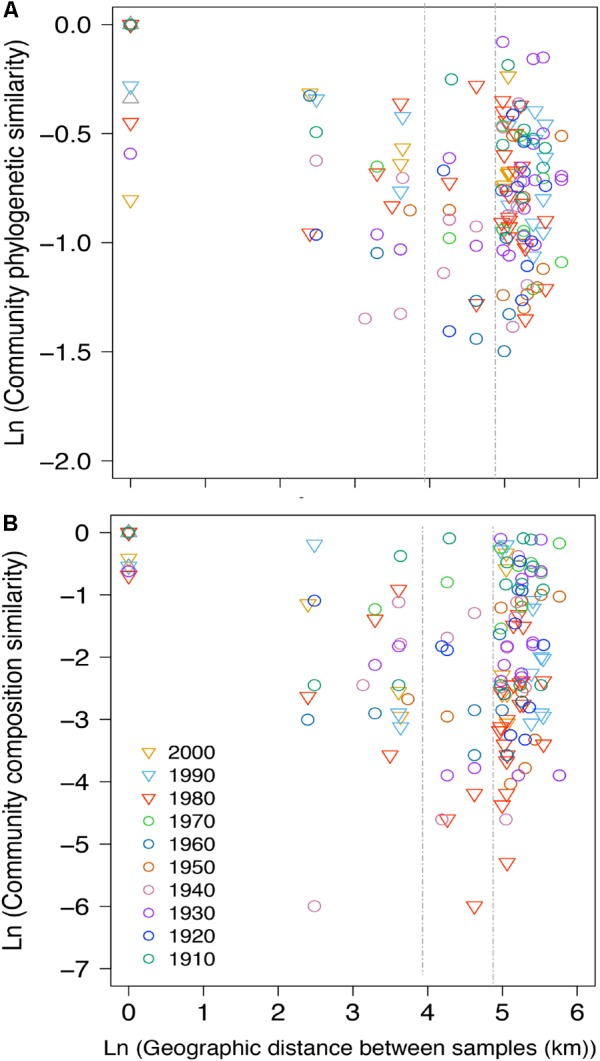
Distance-decay plot showing the natural log-transformed pairwise **(A)** phylogenetic distances (based on Unifrac) calculated among all pairwise communities of NCY and **(B)** community composition similarity (based on Jaccard) at each decade (color-coded) between the 1910s to the 2000s (insufficient pairwise comparisons for the 1900s and 2010s). Each point represents the pairwise natural log-transformed distance between communities plotted against the natural log-transformed geographic distance. A null geographic distance signifies that the pairwise phylogenetic similarity was calculated between samples from the same lake corresponding to the same time-period. The two vertical dashed lines mark 50 and 130 km distances for reference. The absolute geographic distances between lakes (in km) are shown in Supplementary Table S2.

Between-lake phylogenetic similarity (based on Unifrac distances) in NCY communities at each decade between the 1940s and 1970s was mostly stable, with a slight increase starting from the 1980s onward (Figure 5). PERMANOVA, however, did not support a temporal effect over the NCY assemblages during the time span covered by our analysis (i.e., 1940s-2000s; Table 2). Interestingly, no significant discrimination of community similarity was detected in NCY assemblages also north–south of the Alps. PERMANOVA supported a lake effect in phylogenetic community similarity, suggesting a significant lake-specificity of NCY communities (Table 2). The community Unifrac similarity values obtained for each pairwise set of samples plotted against geographic distance revealed no significant distance-decay curve at all time-periods in NCY communities (Figure 6A). The relationship across all pairs of communities at each decade was additionally tested using compositional similarity (Jaccard distances based on presence-absence of OTUs) (Figure 6B). Also in this latter case, there was no significant relationship between compositional similarity in NCY and geographic distance.

**Table 2:** Test of the effects of temporal and spatial factors on community phylogenetic similarities of NCY and photosynthetic cyanobacteria [the latter from Monchamp et al. (2018a,b)], determined by PERMANOVA^∗^.

	Non-photosynthetic cyanobacteria			Photosynthetic cyanobacteria		
	*DF*	*Pseudo-F*	*p*	*DF*	*Pseudo-F*	*p*
Temporal (decades)	8	1.2031	0.211	7	1.7684	**0.001**
Regional (north-south)	1	1.8719	0.1042	1	3.381	**0.0004**
Local (lake)	9	3.6927	**0.0001**	9	2.6669	**0.001**

*p-Values are based on 9,999 permutations, bold face indicates statistical significance (*p* < 0.05), *DF*, degree of freedom. Samples dated to years between the 1940s and the 2010s were used. ^∗^PERMANOVA, Permutational Multivariate Analysis of Variance using Distance Matrices.*

## Discussion

Little is known about the recently discovered clades of NCY in nature. The class Sericytochromatia is the least understood since only three genomes have been sequenced (Soo et al., 2017). The ecological function and the niche occupied by NCY are currently unknown. As reviewed in the introduction, it appears that NCY might be present in both photic and aphotic habitats, with no clear associations between clades and particular environments. Both Melainabacteria and Sericytochromatia have been found in surface waters, including lakes, and algal biofilms (Quast et al., 2013; Gies et al., 2014; Soo et al., 2017; Salmaso et al., 2018). It appears plausible that, like photosynthetic cyanobacteria, Melainabacteria and Sericytochromatia are aquatic life forms. In this study, we found members of NCY at every depth of the sediment cores and in all the peri-Alpine lakes investigated. It could be hypothesized that these organisms are also able to thrive in sediment layers, given their ability to grow under anoxic conditions (Soo et al., 2017). We, however, consider the possibility that this organisms’ habitat is actually the sediment quite unlikely, given the documented presence of NCY in aquatic environments (Table 1). For example, recent evidence suggests that NCY tend to be more prevalent during summer and autumn in the deep, peri-Alpine, and meso-oligotrophic lake Garda (Salmaso et al., 2018). More explorative studies and genome sequencing are needed to shed light on the actual ecology of these bacteria. Here, we consider sediment layers as archives for bacteria living in the lake water and we discuss our findings in terms of changes in NCY assemblages over time.

The topology of our reconstructed phylogenetic tree (Figure 1) reflected the relationships among taxa that were expected from previous work (Di Rienzi et al., 2013; Soo et al., 2014, 2017; Fischer et al., 2016a): we found a clear separation between the Sericytochromatia, Melainabacteria, and photosynthetic cyanobacteria. The number of NCY OTUs recovered from the sedimentary archive of peri-Alpine lakes was much lower in comparison to the richness of photosynthetic cyanobacteria determined previously in the same lakes (Monchamp et al., 2018a). Most of the OTUs were assigned to the deepest-branching paraphyletic group, the Sericytochromatia, and to the Melainabacteria order Gastranaerophilales. As mentioned earlier, the latter OTUs were not retained for further analysis because of their association mostly with guts and feces samples (Di Rienzi et al., 2013; Quast et al., 2013). Gastranaerophilales diversity and distribution in our samples also appears to be purely random, suggesting that their presence in lakes might be the result of release from local point sources, for example via run-off from land, wastewater, or excretions by wild animals and livestock in the lake watershed.

Our results describing the long-term composition and phylogenetic diversity of NCY assemblages in peri-Alpine lakes represent the earliest attempt to explore the distribution of these microorganisms across large spatial and temporal scales, and to investigate their change over broad environmental gradients. Our studied lakes have in fact been characterized by a well-documented history of climate warming and eutrophication, which have led to significant changes in the composition and assembly of photosynthetic cyanobacterial communities (Monchamp et al., 2018a,b). Unlike photosynthetic cyanobacteria, which have increased their diversity at the local and regional scale over the past century (Monchamp et al., 2018a), the richness of environmentally associated NCY did not significantly increase over time, with the exception of Lake Annecy. We have no information to interpret the interesting pattern detected in Lake Annecy. We have no evidence for methodological biases specific for this lake, and neither for directional environmental drivers that are unique to Lake Annecy, which was the most pristine lake in our dataset and it has been impacted by only weak anthropogenic changes over the past century (Monchamp et al., 2018a). No change in community structure, however, suggests that NCY live in a relatively stable lake environment, either in terms of species composition (if they depend on other organisms for growth) or in terms of water physics and chemistry. This is a puzzle, given the evidence for seasonality in NCY dynamics in Lake Garda (Salmaso et al., 2018), and the clear changes in trends and seasonality of lake environments over the past century (Adrian et al., 2009; Smith and Schindler, 2009; Rigosi et al., 2014; Salmaso et al., 2015; Yankova et al., 2017; Monchamp et al., 2018a).

Our results suggest that NCY communities are not shaped by the same environmental drivers as photosynthetic cyanobacteria, and that their composition is most likely lake-dependent (Table 2). We have previously observed that, in the same lakes, the richness and composition of photosynthetic cyanobacterial assemblages were significantly explained by the interaction between warming and eutrophication, with climate change being the strongest driver (Monchamp et al., 2018a,b). PERMANOVA confirmed that photosynthetic cyanobacterial phylogenetic diversity is strongly influenced by temporally changing drivers (Monchamp et al., 2018a,b), and suggest that both local (lake) and regional (north–south) environments play a significant role in shaping composition of these assemblages (Table 2). NCY communities did not show significant temporal dynamics at the decadal scale, and therefore no signs of correlation with temporally changing environmental conditions (Table 2). Similarly, we found no significant effect of regional drivers, like climatic difference between north and south of the Alps (Table 2). We found, however, a significant lake effect on the phylogenetic distance estimated between each pair of NCY communities. Since we did not detect dispersal limitation between lakes, neither for NCY (Figure 6) nor photosynthetic cyanobacteria (Supplementary Figure S2; Monchamp et al., 2018b), our results suggest that local features of lakes are important determinants of community structure in Sericytochromatia and Melainabacteria.

We can only speculate about what the above-mentioned lake features could be. If we assume that NCY thrive in the water-column, as suggested by previous work (Salmaso et al., 2018), it is possible that lake depth, volume, retention time, mixing regime, trophic state and/or catchment characteristics are important factors influencing the composition and diversity of these microorganisms’ communities. Between-lake phylogenetic similarity (based on Unifrac distances) in NCY communities was higher than the average similarity estimated between lakes for photosynthetic cyanobacterial communities (Figure 5; Monchamp et al., 2018a). This could indicate that within-lake factors have, however, a weaker effect on the community composition of NCY than they have on photosynthetic cyanobacterial community change. Understanding of environmental factors driving the diversity and distribution of NCY strongly requires follow up surveys, such as the monitoring of several lakes of different characteristics over depth profiles, and during seasonal succession (Salmaso et al., 2018). It has been suggested that the scale of sampling might be relevant to determine the relative influence of local factors versus environmental gradients (Martiny et al., 2006). Extending our survey of NCY diversity to a larger region might also reveal patterns associated with changes in climate that could not emerge from our peri-Alpine study, or of other environmental gradients that may have played a significant role in the establishment of NCY clades in different lakes and regions. Another important aspect that could not be addressed in our study is the abundance of NCY in lakes, to evaluate their importance for microbial ecosystem functioning.

## Conclusion

Eutrophication and climate warming have been strong environmental drivers during the Anthropocene, impacting lake ecosystem processes (Adrian et al., 2009; Smith and Schindler, 2009) and cyanobacterial community assembly (O’Neil et al., 2012; Rigosi et al., 2014; Huisman et al., 2018; Monchamp et al., 2018a,b). Our study suggests that NCY communities have been unaffected by large-scale anthropogenic environmental change. The lack of decay in phylogenetic similarity over time and over geographic distance across lake communities suggests temporally stable communities with no limitation to dispersal at the regional (peri-Alpine) scale. This is consistent with most reports about microbial dispersal, which rarely showed evidence for geographic distance-decay patterns at the local (0–100 km) and regional (101–5,000 km) scales (Hanson et al., 2012). As previously reported (Monchamp et al., 2018b), the dissimilarity of communities over geographic distance was also non-significant in photosynthetic cyanobacterial assemblages (Supplementary Figure S2). Being temporally stable and lake specific, NCY assemblages could perhaps be used in future studies as an internal lake reference, to which compare patterns of other ecological communities under suspected environmental forcing.

Our study represents an initial exploratory survey of the composition and diversity of ecologically unknown clades of NCY. Our results uncover the diversity of these currently cryptic organisms, which might play a role in lake biogeochemistry. High-throughput sequencing of environmental DNA has the potential to illuminate the ecological niche of these understudied and uncultivable groups of NCY, at the local as well as over large geographic scales. In combination with genome sequencing, diversity surveys and possibly outdoors experiments will help gain understanding on the ecology, evolution and function of these microbes in their natural environment.

## Author Contributions

M-EM, FP, and PS designed the study. M-EM performed the sampling, laboratory work, and data analysis. M-EM and FP wrote the manuscript, which was reviewed and commented by PS.

## Conflict of Interest Statement

The authors declare that the research was conducted in the absence of any commercial or financial relationships that could be construed as a potential conflict of interest.
