# Supplementary material for: Long Term Diversity and Distribution of Non-photosynthetic Cyanobacteria in Peri-Alpine Lakes
Source: Front Microbiol. 2019 Jan 14;9:3344. doi: 10.3389/fmicb.2018.03344 (PMC6340189; doi:10.3389/fmicb.2018.03344)
Supplement: Supplementary file 1 [file Data_Sheet_1.docx]

Supplementary Material

Long term diversity and distribution of non-photosynthetic cyanobacteria in peri-Alpine lakes

Marie-Eve Monchamp, Piet Spaak, Francesco Pomati*

*** Correspondence:** Corresponding Author: francesco.pomati@eawag.ch

**Supplementary Table 1.** List of lakes with their geographic location, main morphological characteristics and trophic status. Data source: Monchamp et al., 2018a, and references therein.

| Lake name | Max depth  (m) | Lake area  (km^2^) | Volume  (10^6^ m^3^) | Elevation  (m a.s.l.) | Latitude | Longitude | Trophic status | *n* samples* |
| --- | --- | --- | --- | --- | --- | --- | --- | --- |
| Annecy | 82 | 27.59 | 1 124.5 | 447 | 45°51′N | 6°10′E | O | 7 |
| Baldeggersee | 66 | 5.2 | 173 | 463 | 47°11′N | 8°15′E | E | 4 |
| Constance  (Upper Lake) | 252 | 472 | 51 400 | 395 | 47°35′N | 9°28′E | M | 7 |
| Geneva | 309 | 582 | 89 000 | 372 | 46°27′N | 6°32′E | M | 5 |
| Greifensee | 33 | 8.5 | 148 | 435 | 47°21′N | 8°41′E | E | 5 |
| Hallwilersee | 47 | 10 | 280 | 449 | 47°17′N | 8°12′E | E | 10 |
| Lugano  (Figino basin) | 288 | 48.9 | 5 860 | 270 | 45°58′N | 08°57′E | E | 9 |
| Maggiore | 370 | 212 | 37 500 | 194 | 45°57′N | 08°38′E | MO | 6 |
| Pusiano | 24 | 5 | 69 | 259 | 45°48′N | 09°16′E | E | 4 |
| Zurich  (Lower Lake) | 137 | 67.3 | 3 300 | 406 | 47°15′N | 8°41′E | MO | 9 |

E; Eutrophic, M; Mesotrophic, MO; Meso-oligotrophic, O; Oligotrophic

* Number of samples used retained after rarefaction and used in diversity analyses.

**Supplementary Table 2.** Pairwise geographic distances between lakes (in kilometers).

|  | LUG | BAL | BOD | HAL | PUS | MAG | ANN | GEN | GRE | ZRH |
| --- | --- | --- | --- | --- | --- | --- | --- | --- | --- | --- |
| LUG | 0 |  |  |  |  |  |  |  |  |  |
| BAL | 147 | 0 |  |  |  |  |  |  |  |  |
| BOD | 183 | 101 | 0 |  |  |  |  |  |  |  |
| HAL | 157 | 10 | 101 | 0 |  |  |  |  |  |  |
| PUS | 22 | 169 | 200 | 179 | 0 |  |  |  |  |  |
| MAG | 26 | 142 | 192 | 151 | 41 | 0 |  |  |  |  |
| ANN | 217 | 220 | 317 | 222 | 229 | 191 | 0 |  |  |  |
| GEN | 195 | 156 | 256 | 157 | 212 | 171 | 72 | 0 |  |  |
| GRE | 155 | 36 | 65 | 37 | 175 | 156 | 255 | 192 | 0 |  |
| ZRH | 144 | 32 | 70 | 36 | 165 | 145 | 248 | 187 | 11 | 0 |

**Supplementary Table 3.** Summary of the number of samples retained, total number of OTUs, and OTU richness per samples at three rarefaction depths.

| Rarefaction depth (number of reads) | *n* samples retained | Total number of OTUs | OTU richness/sample (range) |
| --- | --- | --- | --- |
| 201 | 66 | 63 | 2-19 |
| 500 | 40 | 52 | 2-19 |
| 1000 | 31 | 47 | 3-16 |
|  |  |  |  |

## Supplementary Figures


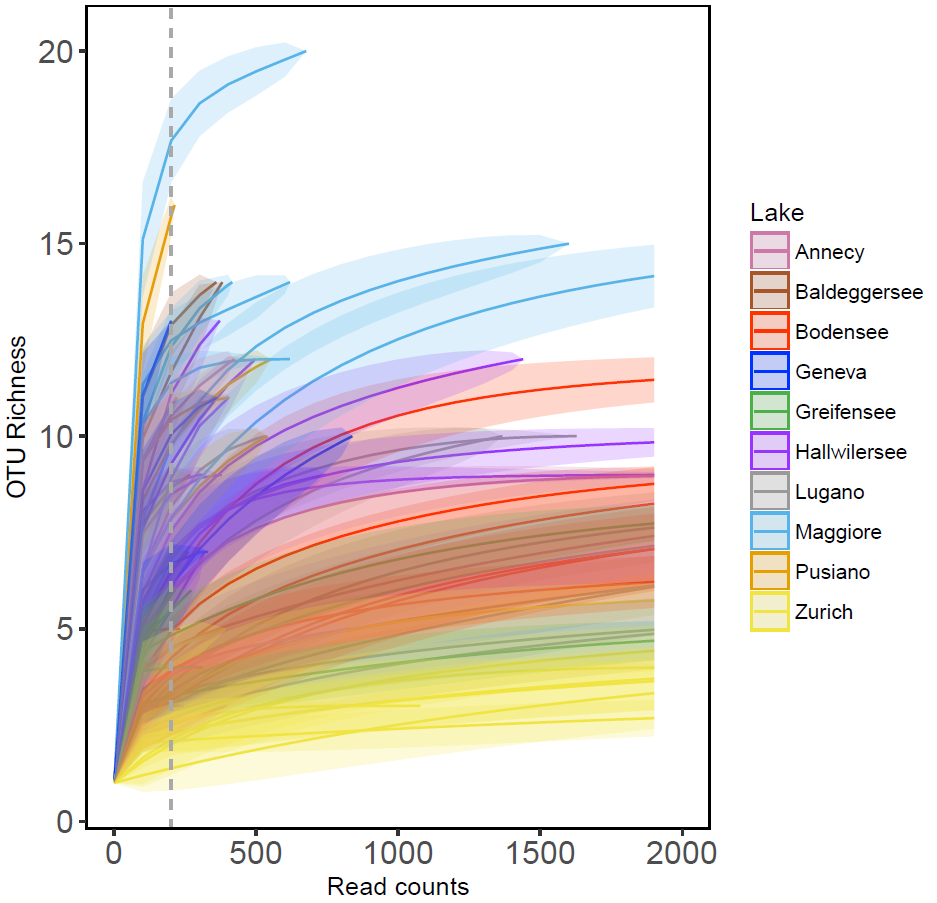


**Supplementary Figure 1.** Rarefaction plot of all the non-photosynthetic cyanobacteria samples from the ten lakes (read count limit set at 2000 for visualization). The sequencing coverage was variable among samples and across lakes. There is no visible effect of time on the sequencing depth that would indicate a loss of richness due to DNA degradation with sediment age. Based on this plot and the information summarized in table S3, we decided to rarefy the samples to 201 reads in order to retain as many samples as possible for the analysis and considering that the asymptote is reached in most samples at this sequencing depth.

**
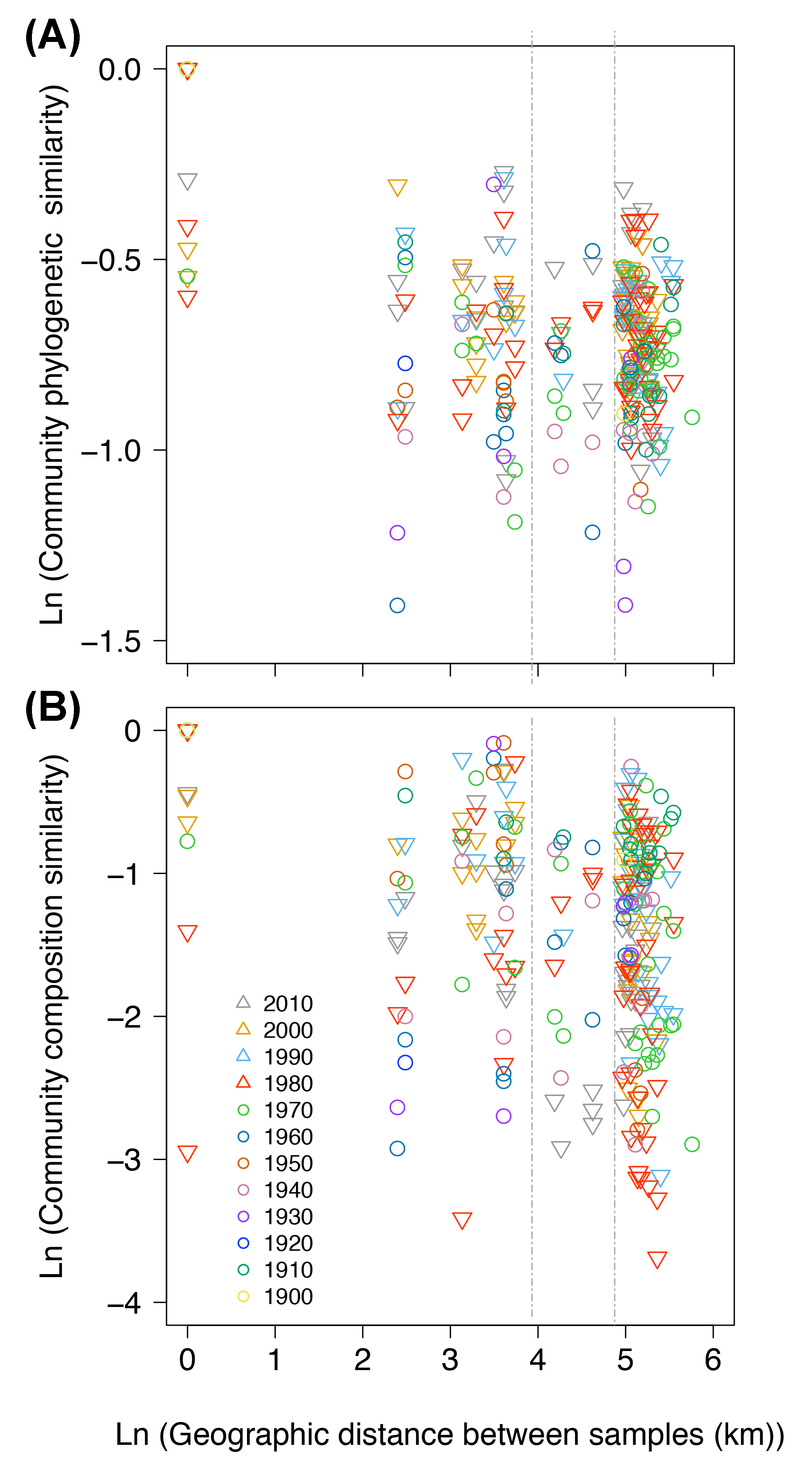
**

**Supplementary Figure 2.** Distance-decay plot showing the **(A)** natural log-transformed pairwise phylogenetic distance (based on Unifrac) and **(B)** natural log-transformed pairwise taxonomic distances (based on Jaccard similarity on incidence of OTUs) calculated among all communities of **photosynthetic cyanobacteria** at each decade between the 1900s and the 2010s. Each point represents the pairwise distance between communities plotted against natural log-transformed geographic distance. A geographic distance of 0 signifies that the pairwise similarity was calculated between samples from a same lake at a given time period. In opposition to NCYs for which no significant distance-decay relationship was detected, the photosynthetic cyanobacterial communities showed significant decrease in similarity along a distance gradient at five time periods: 1950s, 1970s, 1990s, 2000s, and 2010s. The two vertical dashed lines mark distances of 50 km and 130 km for reference.
